# Physiological Variables, Milk Conductivity and Production in Dairy Cows to Ketosis During the Transition Period in Northern Mexico

**DOI:** 10.3390/vetsci13070622

**Published:** 2026-06-26

**Authors:** Pedro Antonio Robles-Trillo, Christopher D. Lu, Luis Jesús Barrera-Flores, Rafael Rodríguez-Venegas, Martín Alfredo Legarreta-González, Rafael Rodríguez-Martínez

**Affiliations:** 1 Departamento de Ciencias Médico Veterinarias, Universidad Autónoma Agraria Antonio Narro, Unidad Laguna Periférico Raúl López Sánchez S/N, Torreón CP 27054, Coahuila, Mexico; parobles58@gmail.com (P.A.R.-T.); rafar.v.v@gmail.com (R.R.-V.); 2 College of Agriculture, Forestry and Natural Resource Management, University of Hawaii, Hilo, HI 96720, USA; chrislu@hawaii.edu; 3 Facultad de Agricultura y Zootecnia, Universidad Juárez del Estado de Durango, Carretera Gómez Palacio a Tlahualilo km 32, Gómez Palacio CP 35000, Durango, Mexico; luis.barrera@ujed.mx; 4 Tecnológico de Monterrey, Escuela de Ingeniería y Ciencias, Ave. Eugenio Garza 2501 Sur, Col. Tecnológico, Monterrey CP 64700, Nuevo León, Mexico

**Keywords:** exploratory factor analysis, ketosis, dairy cows, activity time, ruminating time, milk electrical conductivity

## Abstract

Attempting to detect and improve the management of Ketosis, the objective of this study was to determine and confirm the relationship between hours of activity, rumination time, conductivity, and milk production with the presence of ketosis in cows during the transition period in dairy cows in the Comarca Lagunera region, the heart of the dairy cattle production in Mexico. Data were collected in a large scale dairy cattle study. High-precision electronic collar sensors, high-precision electronic scales, and online electronic weighing sensors were employed to determine activity and ruminating time, milk electrical conductivity, and milk yield, respectively. All data were collected and integrated using an electronic peripheral management and control software. Using urinary ketone bodies measured by qualitative strips as the biomarker for ketosis, 10.50% of the cows were found to be positive for ketosis, while the remaining 89.50% were negative. The mean and standard error for activity time (AT), ruminating time (RT), milk electrical conductivity (CE) and milk yield (MY) in normal (N) vs ketotic (P) cows were: AT N 61.38, ± 0.39, AT P 39.08 ± 0.49; RT N 530.85 ± 2.94, RT P 295.24 ± 10.69; CE N 5.68 ± 0.03, CE P 9.13 ± 0.11; and MY N 38.87 ± 0.29, MY P 20.34 ± 0.54. Exploratory Factor Analysis (EFA) was conducted for the purpose of uncovering the underlying structure of the data by identifying latent constructs that influence the observed variables. The EFA estimated two factors which explained 62% of the variation observed. The Factor 1 (MR1) comprising the variables MY and EC, and Factor 2 (MR2), which consists the variables AT and RT. High-precision measurement sensors along multivariable analyses could facilitate the establishment of a correlation between ketosis and variables associated with the physiology, well-being, and productivity of bovines in the transition period. It further open the possibility of early detection of metabolic diseases such as ketosis.

## Simple Summary

Physiological variables associated ketotic cows were recorded, analyzed and confirmed using sensors along with electronic peripheral management and control software. It opens the possibility for early detection and improved management of ketosis in large scale dairy cattle production.

## 1. Introduction

The transition period from late gestation to the onset of lactation is subject to hormonal, metabolic and behavioural changes, during which more than 90% of diseases occurred [1]. This period, which occurs between 21 days before calving and 21 days after calving, is a stage in which dry matter intake is crucial for maintaining an adequate metabolic, productive and reproductive responses [2]. However, during this period, an increase in the mobilisation of non-esterified fatty acids has been observed, which has led to a decrease in dry matter (DM) consumption and subsequent reduction in milk production [3].

The absence of adequate nutrition in livestock has been demonstrated to result in the onset of metabolic diseases and a decline in overall body condition. This, in turn, has been shown to lead to diminished reproductive and productive efficiency [4]. Therefore, the early detection and proactive management of ketosis can minimise the reduction in milk production and contribute to a more sustainable dairy production [5]. Ketosis is a salient condition arising from a metabolic imbalance resulting from a lack of DM consumption and, consequently, a lack of glucose supply in the cow’s attempt to recover from the energy imbalance. The imbalance under discussion has been shown to result in elevated levels of ketone bodies (beta-hydroxybutyrate, acetoacetate and acetone). These have been demonstrated to impact liver function and metabolism, giving rise to manifestations derived from primary (subclinical) or secondary (clinical) ketosis. These affect the health, productivity and reproductive performance of the cow, as well as the profitability of the farm [6]. As demonstrated in the research by Cainzos et al. [7] and Rico and Barrientos-Blanco [8], the phenomenon is associated with economic and productive losses that result in the premature removal of animals from farms.

As demonstrated in the study by Itle et al. [9], clinical ketosis and standing behaviour have been reported in transition dairy cows. As posited by Goldhawk et al. [10], the mean total daily standing time was found to be significantly greater in clinically ketotic cows than in non-ketotic cows during week −1 and day 0 relative to calving. As demonstrated by Itle et al. [9], the prevalence of standing for extended periods was found to be more significant in clinically ketotic cows than in non-ketotic cows. Furthermore, rumination in dairy cattle can serve as a significant indicator of metabolic health. Monitoring the duration of rumination is considered to be among the most efficacious methods of identifying subclinical ketosis, whereby the duration of eating, chewing, and ruminating can act as biomarkers [11]. In order to achieve this objective, the implementation of pedometers (leg and neck responders) in conjunction with rumination recording collars enables the estimation of behavioural patterns, ruminal activity, motor activity, and neck position, which are concomitant with the duration of feed consumption by bovines [12]. Kaufman et al. [13] hypothesises that rumination during the transition period may contribute to the identification of subclinical ketosis and other health problems in multiparous cows.

The advent of high-precision technology has facilitated the acquisition of data on livestock behaviour, thereby enabling informed decision-making regarding livestock management in intensive conditions. These technologies are utilised for their capacity to monitor and measure production, physiological, or reproductive variables in animals, with the objective of enhancing management strategies and overall efficiency [14]. Automated milking systems that provide data on milk production and the electrical conductivity of each animal’s milk at each milking are a constant source of information about each animal. It has been demonstrated that both measurements are sensitive to changes in the animals’ health status, especially changes in milk electrical conductivity [15]. However, various limitations are in place that hinder the implementation of these technologies within the domain of dairy production systems. In this regard, Lamanna et al. [16] have reported that the primary constraints on effective adoption are substantial installation costs and suboptimal compatibility between systems. The advantages offered by these systems position the dairy industry at a pivotal juncture, characterised by the imperative to establish digital frameworks that can effectively address the intricacies of animal biology, the necessity for environmentally sustainable productivity, and the societal expectations of environmental responsibility [17].

Concurrent with these technological advancements has been the evolution of data analysis. In this regard, exploratory factor analysis (EFA) is a statistical method used to identify underlying variables, or factors, that explain the pattern of correlations within a set of observed variables. The objective of this method is twofold: firstly, to explore and establish an internal structure by generating new factors from a given set of variables; secondly, to reduce the number of these variables. The objective of this study was twofold: firstly, to measure the prevalence of ketosis in cows that were either positive or negative for ketosis during the transition period in three barns in the Comarca Lagunera region; and secondly, to determine the relationship between hours of activity, rumination time, conductivity and milk production using EFA.

## 2. Materials and Methods

### 2.1. Study Location

The study was conducted using data generated in three barns located in the municipality of Tlahualilo de Zaragoza, Durango, 25°52′39″ LN 103°23′39″ LO, at an altitude of 1126 masl. The mean annual rainfall is 300 mm, while the mean annual temperature is an average annual temperature of 21 °C, ranging from 0 °C to 37 °C [18].

### 2.2. Study Animals

The animals selected for inclusion in the study comprised 486 cows of the Holstein breed, ranging in lactation from two to four, with an average of 3.1 lactations and a mean milk yield of 11,959 L during the previous lactation. The cows were fed a completely mixed diet, with a range of six to eight feedings per day in each pen of the transition cow groups. The diets according to the nutritional requirements established by the National Research Council of National Academies of Sciences, Engineering, and Medicine [15]. The diets utilised forage at a percentage range of 40% to 60% and concentrate at a percentage range of 60% to 40 %, with an approximate dry matter (DM) percentage of 50%. The diets were prepared in total mixed Rations (TMR) mixer wagons, with a capacity of 10,000 kg. On all three farms, the cows were milked thrice daily.

The management of livestock is consistent with the standards of field studies that reflects the scientific and practical aspects of livestock husbandry, including the breeding, feeding, and care of animals used for food, fibre, or other agricultural products. All farms were in compliance with the established comfort standards and the animals were under the supervision of a qualified veterinarian [19].

### 2.3. Variables Analysed

The activity time (AT) and ruminating time (RT) of the cows in the pen was determined using high-precision electronic collar sensors from Alta Cow Watch & Nedap (NEDAP GmbH, Rulo, The Netherlands), which record daily activity units per cow. Milk Electrical conductivity (MEC) was measured using high-precision Metatron Demas electronic scales (Westfalia-Surge GmbH, Oelde, Germany) that record the number of mS/cm, with the milk being mixed from the four quarters. The quantity of milk produced (MY, in kg) was measured using Metatron Demas online electronic weighing sensors (Westfalia-Surge GmbH, Oelde, Germany). Using high-precision automated systems in the pens, all data were recorded in the DAIRY PLAN DP 21 electronic peripheral management and control software (Westfalia-Surge GmbH, Oelde, Germany). The presence of ketone bodies (KB) in urine was determined through the utilisation of urine test strips (Keto-Diastix, Bayer, Whippany, NJ, USA). The interpretation of the results obtained from the use of the strips is conducted within the context of four distinct levels: (1) 0–5 mg/dL (negative); (2) 15 mg/dL (small, positive, low); (3) 40 mg/dL (positive, moderate); (4) >40 mg/dL to 160 mg/dL (positive, large). For the purposes of this investigation, the term ‘positive cows’ was defined as ‘ketosis’ from the second level onwards. It should be noted that all the variables (AT, RT, EC MY and KB) were measured on the 5th day postpartum [20]. Data collection period ran from May to September 2025.

### 2.4. Statistical Methods

An Exploratory Factor Analysis (EFA), was conducted for the purpose of uncovering the underlying structure of the data by identifying latent constructs that influence the observed variables.

The decision to undertake these particular analyses was motivated by several factors. The analyses are chiefly concerned with facilitating data reduction by means of simplifying the database through the grouping of variables into a smaller number of factors. Secondly, it identifies patterns that reveal relationships between variables. Thirdly, it validates constructs, thereby assisting in the testing or development of measurement instruments. Moreover, the versatility of the analyses is attributable to its application across various disciplines [21]. The proposed approaches serve to enhance interpretability by consolidating redundant variables, thereby facilitating improved understanding. Following the identification of the factors, the correlation between them was measured.

The Kaiser–Meyer–Olkin (KMO) index was utilised in the analysis. As described in Ref. [22], the KMO index is a statistical test employed in factor analysis to ascertain the suitability of the data for this process. KMO is a statistical technique that is employed to assess the sampling adequacy of each observed variable within a model, as well as the overall adequacy of the model as a whole. Furthermore, the sphericity of the data was assessed through the implementation of Bartlett’s test.

In order to ascertain the number of factors, a parallel analysis was conducted. This analysis constitutes a simulation-based method that generates random data exhibiting characteristics analogous to the observed data. The subsequent stage of the process involves the comparison of the eigenvalues extracted from the observed data with those from the simulated data.

We used R [23] and the R-packages car (Version 3.1.3; [24]), carData (Version 3.0.5; [25]), dplyr (A Grammar of Data Manipulation, Version 1.1.4; [26]); emmeans (Version 2.0.1; [27]), forcats (Version 1.0.1; [28]), ggplot2 (Version 4.0.1; [29]), lubridate (Version 1.9.4; [30]), pander (Version 0.6.6; [31]), papaja (Version 0.1.4; [32]), purrr (Version 1.2.1; [33]), r2glmm (Version 0.1.3; [34]), readr (Version 2.1.6; [35]), readxl (Version 1.4.5; [36]), stringr (Version 1.6.0; [37]), tibble (Version 3.3.1; [38]), tidyr (Version 1.3.2; [39]), tidyverse (Version 2.0.0; [40]), EFAtools (Version 0.7.1; [41]) and tinylabels (Version 0.2.5; [42]) for all our analyses.

## 3. Results

Table 1 presents the arithmetic mean, along with the standard error (se), stratified by negative and positive cows to ketosis of the variables AT, RT, MY and EC. In addition, the investigation revealed that 10.50% of the bovines were identified as positive for ketosis, while the remaining 89.50% were negative (*p* = 0.001, $\chi^{2}$ = 303.41, df = 1).

### Exploratory Factor Analysis

**Figure 1:**
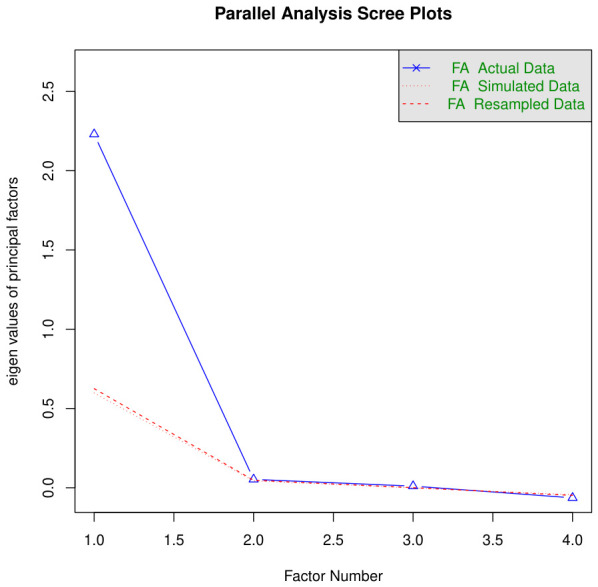
Parallel analysis scree plot.

The overall KMO value was 0.79 and the values for each variable are: AT, 0.85; RT, 0.78; MY, 0.81; and EC 0.74. The overall KMO value of the data is meritorious; thus, these data are probably suitable for factor analysis. Bartlett’s test of sphericity was significant at an alpha level of 0.05, and thus, these data are probably suitable for factor analysis ($\chi^{2}$ (6) = 733.12, *p* < 0.001). The results for the test of the hypothesis that two factors are sufficient were $\chi^{2}$ (6) = 733.12, *p* < 0.001. Furthermore, a parallel analysis was conducted in order to verify the results graphically, as demonstrated in Figure 1.

The total variability explained by the standardised loadings (pattern matrix) based upon correlation matrix is 62% (31% by MR1 and 31% by MR2).

A reliability analysis was conducted. In order to assess the reliability of these factors, the standard Cronbach’s alpha value was employed. This resulted in a value of 0.83, which, according to the accepted criteria, indicates a good reliability estimate.

**Figure 2:**
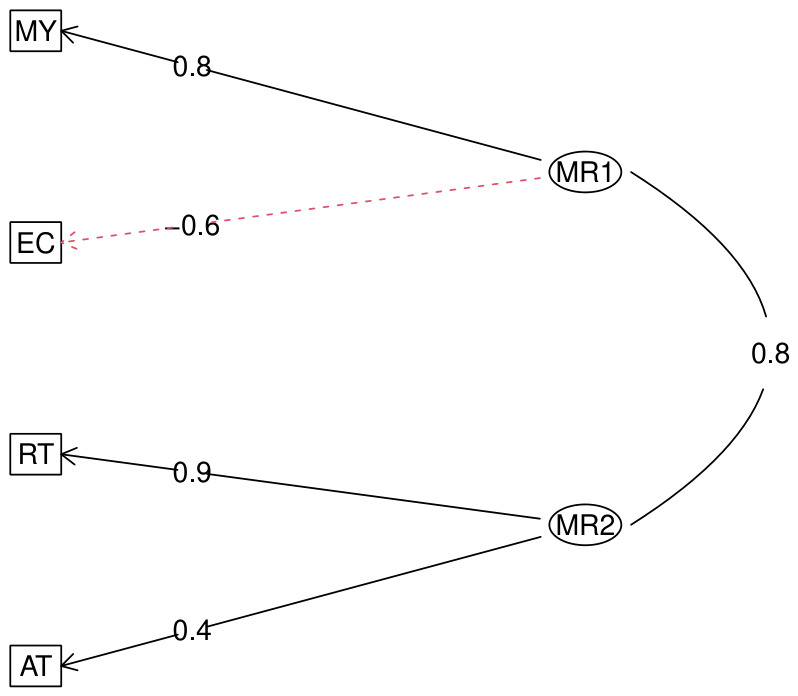
Exploratory Factor Analysis for Activity time (AT), milk produced (MY), ruminating time (RT), electrical conductivity (EC), Factor 1 (MR1), and Factor 2 (MR2) in cows on the 5th day of lactation, whether or not they were positive for ketosis, on an intensively managed farm in northern Mexico. A positive correlation is indicated by black solid lines, while a negative correlation is indicated by red-dotted lines.

The EFA helped to identifying underlying factors. This analysis yielded two factors. The first factor (MR1) encompasses the variables MY and EC, while the second factor (MR2) comprises the variables AT and RT, as illustrated in Figure 2. Furthermore, the figure indicates a correlation of 0.79 between the two factors.

## 4. Discussion

The present study proposes a methodological approach that diverges from conventional practices, adopting a holistic perspective to analyse the variables associated with the occurrence of ketosis in dairy cows during the transition period. This approach entailed the replacement of the conventional, individual variable analysis with a more comprehensive integrated methodology. The objective of the methodology was to identify the association between ketosis and yield, as well as conductivity of milk and activity of cows. The measurement of these variables was undertaken with the aid of high-precision instrumentation.

### 4.1. Ketosis Prevalence

The results of the study indicate a prevalence of 10.5% for ketosis in the cows included in the study. With regard to the available data, it is noteworthy that the prevalence obtained is low, as evidenced by reports of an overall prevalence of subclinical ketosis in 10 European countries of 21.8%, ranging from 11.2% to 36.6%, with a higher prevalence observed in cows between 2 and 15 days in milk (DIM) [43]. In a 2022 meta-analysis, Loiklung et al. [44] reported an overall prevalence of subclinical ketosis in Holstein cows of 22.7%, with no significant differences observed in the prevalence of subclinical ketosis between continents, diagnostic techniques, sample types (milk and blood) or number of calvings. However, the prevalence in Holstein cows (19.8%) was significantly lower than in other mixed breeds (23.7%). Furthermore, Kemel et al. [45] posits that while clinical signs of ketosis are observed in 2–15% of bovines, the overall subclinical prevalence is substantially higher (22.7%), and is often underestimated by farmers. The low prevalence observed in our study can be attributed to the fact that only recently calved cows (i.e., five days into lactation) were considered in the analysis. This phenomenon can be attributed to the fact that farm practice involves the testing of all cows for ketosis on the fifth day of lactation; consequently, the incorporation of the remaining subjects in the herd may yield divergent outcomes and elevate the prevalence.

### 4.2. Exploratory Factor Analysis (EFA)

The EFA identified two factors associated with ketosis in the bovines under scrutiny in the present study; furthermore, MR1 and MR2 exhibit an association coefficient of 0.76, indicating a significant relationship between the two factors (Figure 2). The ‘parallel analysis scree plot’ was employed to estimate the number of factors for the analysis. In this instance, the scree plot yielded two factors. Furthermore, the results of the test of the hypothesis that two factors are sufficient were statistically significant ($\chi^{2}$ (6) = 733.12, *p* < 0.001).

#### 4.2.1. MR1 Factor

MR1 has been found to be associated with milk yield, exhibiting a positive correlation coefficient of 0.8, and with the electrical conductivity of milk, displaying a negative correlation coefficient of −0.6. In this regard, the mean milk production of cows testing positive for ketosis was 20.3 kg, whilst those testing negative averaged 38.9 kg. This figure indicates a 44.7% decrease in milk production among the former. This finding is consistent with the observations reported by Tsai et al. [46], who noted that cows experiencing ketosis exhibited a decline in milk production, thereby suggesting a correlation between ketosis and milk production. As demonstrated by Antanaitis et al. [47], subclinical ketosis has been shown to result in a decline in milk production from day −6 to the day of diagnosis. In the study conducted by Taechachokevivat et al. [48], the potential of long short-term memory (LSTM) models to identify ketosis was demonstrated. The utilisation of standard deviations in milk production, variations in rumination time and activity index relative to the median according to the number of calvings and DIM, with different time series (lengths and number of days before diagnosis), served to illustrate this potential. However, it is noteworthy that no commercially available health alert system employs LSTM models or utilises deviations from the herd median, adjusted for herd-specific number of calvings and DIM, as input variables.

The relationship between milk production and ketosis has demonstrated equivocal outcomes, contingent on the number of days in milk. Specifically, it has been observed to be negative when subclinical ketosis is identified at the onset of lactation and has been linked positively or negatively when subclinical ketosis is diagnosed postpartum. The outcomes of interest are influenced by the cows’ calving history [49], which determines the establishment of future variables of interest. These variables are to be related to the records of calvings.

Furthermore, the results of the present study demonstrated a correlation between BK and milk conductivity. This finding is consistent with the results reported by Antanaitis et al. [47], who observed that electrical conductivity in cow’s milk increased more significantly two days before the onset of clinical symptoms of ketosis (3.1–3.7%). The correlation between both factors (MR1 and MR2) demonstrates a positive association (0.76) (Figure 1), thus suggesting a reciprocal relationship between the two factors; i.e., an increase in one will be accompanied by an increase in the other. In comparison with healthy cows, cows with ketosis generally exhibited reduced milk production and a decreased rumination duration per day [50,51].

With regard to EC, a moderate factor load (of −0.6) was demonstrated. It is considered beneficial to establish a baseline for each individual cow, given the high level of variation that is observed among animals. In addition to the sensor utilised in this study, automatic monitoring systems such as the RumiWatch noseband sensor [52] or the AfiCollar [53] can prove advantageous in achieving this objective. These systems possess the capacity for real-time monitoring, thereby enabling the collection of pertinent data. It is imperative to detect subclinical ketosis before the onset of clinical symptoms. The hypothesis that low rumination cows may benefit from treatment with propylene glycol has been proposed [54], while those with high rumination may naturally recover. A substantial decline in rumination, spanning between 12 and 24 h, has been identified as a reliable indicator of imminent ketosis. Consequently, the assessment of blood ketones, including BHB and NEFA, is important.

The integration of behavioural monitoring with milk analysis, as exemplified by the system utilised in this study or DeLaval’s Herd Navigator [55], has been demonstrated to be a valuable approach for the identification of SCK. An increase in milk conductivity concomitant with a reduced milk yield can be observed in SCK cows. As Antanaitis et al. [47] demonstrate, milk conductivity is frequently utilised as an indicator of energy imbalance and metabolic stress in dairy cows with mastitis. The conductivity exhibited a reduction two days prior to the onset of clinical ketosis.

#### 4.2.2. MR2 Factor

The MR2 factor was found to be associated with cows that were negative for ketosis, and this association was found to be related to rumination time and milk production. The relationships between MR2 and its variables demonstrate a relationship with AT at a factorial load of 0.9 during the rumination period, which is considered to be a very strong factorial load of ketosis, and of 0.4 with the activity time of cows. This finding is consistent with the observations reported by Antanaitis et al. [47], who noted that the activity (steps per hour) of cows diagnosed with ketosis during the three days prior to calving was 9.8 % higher than that of healthy cows. Antanaitis et al. [56] hypothesised that there is a relationship between minutes of locomotor activity and the presence of ketosis. The hypothesis posits that the walking behaviour of sick cows is subject to a decrease, with a reduction in minutes of walking being observed from 13 days before the onset of the disease to the day of onset. This decline is in contrast to the walking behaviour of healthy cows, which remains consistent over the same period. Furthermore, as Tsai et al. [46] observed, cows in a state of ketosis demonstrated reduced levels of activity and decreased rumination time.

Rutherford et al. [57] observed that physical activity around oestrus is reduced by ketosis in early lactation. However, this negative effect appears to diminish as the cow’s lactation progresses. In the study conducted by Najm et al. [58], a significant reduction in the motor activity of cows diagnosed with ketosis from day 6 to day 12 postpartum was observed when compared to healthy cows (*p* < 0.001, $\chi^{2}$ test). This finding suggests the potential for a significant association between motor activity and group affiliation, as evidenced by the results of the logistic regression models. Kaufman et al. [13] posited that multiparous cows afflicted with ketosis exhibited a tendency to maintain a recumbent posture for a more protracted period when compared to their healthy multiparous counterparts during the postpartum phase. As Vasseur et al. [59] demonstrated, first-calf cows with ketosis exhibited a higher frequency of resting episodes and shorter resting episodes than multiparous cows. Furthermore, an increase in the average duration of these resting episodes was observed as the number of days in milk increased. It has been established that alterations in standing activity occur prior to the initial manifestations of clinical ketosis [58]. Consequently, a timeframe of four to five days might prove advantageous for the earlier detection of such changes.

As demonstrated by Kaufman et al. [13], the mean duration of ruminating for bovines that were negative for ketosis was 459.00 ± 11.3 min. In contrast, the present study demonstrated that bovines experiencing ketosis exhibited a significant reduction in ruminating time, with an average decrease of 25 to 48 min from week −1 one week prepartum to one week postpartum. The mean of 530.85 units in negative cases, as revealed by the results of the present study, is significantly lower than the 295.24 units recorded in positive cases. This finding indicates a 44.3% decrease in the time spent ruminating. Antanaitis et al. [47] reported that healthy cows exhibited a longer duration of rumination per day than those that developed ruminal acidosis and ketosis (4.3 and 13% longer, respectively). As Antanaitis et al. [56] also reported, rumination was found to be reduced in SCK cows 17 days prior to the diagnosis of ketosis from a cow fitted with a sensor. In a subsequent study with a larger number of cows, a clear trend of reduced eating and ruminating times in SCK cows was established [60]. As demonstrated by Kaufman et al. [13], there was a gradual decrease in rumination time from day −6 to calving in both healthy cows without BK and cows with BK. However, the decrease was more pronounced in cows diagnosed with BK alone than in cows with other conditions, with cows with ketosis ruminating 25 to 44 min less during that period. The decline in RT observed in cows positive for ketosis may be attributable to reduced intake and/or alterations in the fermentation pattern within the rumen [61].

### 4.3. Limitations of the Study

In accordance with the farm’s policy, we were only granted access to data from the ketosis test carried out on the farm itself. The test employed test strips to measure ketone bodies in urine, a method that may be less accurate than blood tests. Furthermore, the study did not encompass data pertaining to additional variables, such as the quality of milk and the occurrence of ketosis in animals during various lactation periods. Furthermore, it is imperative to acknowledge the intricacy inherent in the interpretation of behavioural data in real farm settings, where a multitude of overlapping factors exert a profound influence on sensor outputs. These factors must be meticulously assessed in forthcoming studies.

## 5. Conclusions

The present study demonstrated that the utilisation of high-precision measurement sensors could facilitate the establishment of the association between ketosis and several variables associated with the physiology, well-being and productivity of bovines in the transition period. Furthermore, the employment of high-precision technology for data collection signifies a promising avenue for the prediction of metabolic diseases in dairy cows. The utilisation of a high-precision sensor has the potential to facilitate the establishment of an individual animal baseline, thereby minimising variations among animals and contributing to the early detection of metabolic diseases, such as ketosis. Furthermore, the present study demonstrates the efficacy of exploratory factor analysis as a tool for analysing health issues in dairy cattle. This objective is realised through the implementation of a holistic approach, as opposed to a pairwise variable comparison.

## Figures and Tables

**Table 1:** Mean and standard error for negative and positive cows to ketosis for activity time, ruminating time, milk electrical conductivity and milk yield.

	Mean	se
Activity time (units)		
Negative	61.38	0.39
Positive	39.08	0.49
Ruminating time (units)		
Negative	530.85	2.94
Positive	295.24	10.69
Milk yield (kg)		
Negative	38.87	0.29
Positive	20.34	0.54
Milk electrical conductivity (mS/cm)		
Negative	5.68	0.03
Positive	9.13	0.11

## Data Availability

Please note that the data set used in this article is not currently available, as it is owned by the dairy company that provided it. Should you wish to access the data set, please direct your request to Rafael Rodríguez Martínez (rafael.rdz.mtz@gmail.com).

## Abbreviations

The following abbreviations are used in this manuscript:

DM	Dry matter
EFA	Exploratory factor analysis
TMR	Total mixed ration
AT	Activity time
RT	Ruminating time
MEC or EC	Milk electrical conductivity
MY	Milk yield or milk produced
MR	Factor
